# No effect of user fee exemption on perceived quality of delivery care in Burkina Faso: a case-control study

**DOI:** 10.1186/1472-6963-14-120

**Published:** 2014-03-11

**Authors:** Aline Philibert, Valéry Ridde, Aristide Bado, Pierre Fournier

**Affiliations:** 1 Biology Department, University of Ottawa (UdO), 325 MacDonald Hall, 150 Louis Pasteur, Ottawa, ON K1N 6 N5, Canada; 2 Centre de recherche du CHUM (CRCHUM) and School of Public Health, Montréal, Canada; 3 Institut de Recherche en Sciences de la Santé (IRSS) et Centre National de la Recherche Scientifique et Technologique (CNRST), Ouagadougou, Burkina Faso

**Keywords:** Fee exemption, Primary health care, Birth delivery, Patient’s satisfaction, Burkina Faso

## Abstract

**Background:**

Although many developing countries have developed user fee exemption policies to move towards universal health coverage as a priority, very few studies have attempted to measure the quality of care. The present paper aims at assessing whether women’s satisfaction with delivery care is maintained with a total fee exemption in Burkina Faso.

**Methods:**

A quasi-experimental design with both intervention and control groups was carried out. Six health centres were selected in rural health districts with limited resources. In the intervention group, delivery care is free of charge at health centres while in the control district women have to pay 900 West African CFA francs (U$2). A total of 870 women who delivered at the health centre were interviewed at home after their visit over a 60-day range. A series of principal component analyses (PCA) were carried out to identify the dimension of patients’ satisfaction.

**Results:**

Women’s satisfaction loaded satisfactorily on a three-dimension principal component analysis (PCA): 1-provider-patient interaction; 2-nursing care services; 3-environment. Women in both the intervention and control groups were satisfied or very satisfied in 90% of cases (in 31 of 34 items). For each dimension, average satisfaction was similar between the two groups, even after controlling for socio-demographic factors (p = 0.436, p = 0.506, p = 0.310, respectively). The effects of total fee exemption on satisfaction were similar for any women without reinforcing inequalities between very poor and wealthy women (p ≥ 0.05). Although the wealthiest women were more dissatisfied with the delivery environment (p = 0.017), the poorest were more highly satisfied with nursing care services (p = 0.009).

**Conclusion:**

Contrary to our expectations, total fee exemption at the point of service did not seem to have a negative impact on quality of care, and women’s perceptions remained very positive. This paper shows that the policy of completely abolishing user fees with organized implementation is certainly a way for developing countries to engage in universal coverage while maintaining the quality of care.

## Background

Concerned with the consequences of user fees in African countries, in 2010 the African Union decided to remove user fees in order to enhance access to maternal health care [1]. Indeed, user fee strategies, introduced since the 80s, have generally been shown to impede access to health care, particularly for poor and vulnerable population groups that failed to meet health care costs [2-4]. Fees have been pointed out as contributing to the impoverishment of vulnerable households and increasing pre-existing inequalities [5,6]. Following an emerging international consensus on the removal of user fees, some African countries have adopted a fee abolition policy [7,8]. The resulting growing utilization of health care facilities for deliveries after partial or total fee exemption [9,10] has been accompanied by a significant loss of income for health care facilities. To compensate for this lack of income while ensuring the maintenance of services and anticipating growth in demand, several health care facilities have reduced the quality of care services and/or reinstated payment through informal practices [11-15]. Decreased quality in delivery care services may be the result of increased workload or declining staff morale, particularly when the implementation of the exemption policy is not accompanied by a commensurate increase in human, material and financial resources [16]. This type of decline in care service quality may cause frustration among patients [17], especially the poorest women, who usually suffer poor treatment, as health workers are likely to give preference to wealthy women who offer tips and bribes [18]. Although many studies have centered on care providers’ satisfaction in Africa [19-21], little emphasis has been placed on patients’ satisfaction in the new context of elimination of user fees [22].

Since 2006, Burkina Faso has implemented a national subsidy policy that covers 80% of fees for skilled deliveries in primary health care centres (Health and Social Promotion Centres, or CSPS), but the remainder must still be paid by the women [23]. In 2008, the German non-governmental organization (HELP) introduced a supplementary subsidy (20%) in the health districts of Sebba and Dori, thus ensuring totally free care at the point of use. It remains be to verified whether quality of care was maintained for everyone after total removal of fees. Thus, the present study attempts to gather evidence on satisfaction with quality of care of skilled delivery for women who benefited from total fee exemption and those who did not (still paying 20% of fees).

## Methods

### Intervention

In September 2008 HELP launched a humanitarian intervention to cover total fees of delivery care by eliminating the remaining 20% of user fees in some health districts in the Sahel region of Burkina Faso. For this, HELP gave the CSPS a subsidy equal to the women’s share of the cost, up to 900 West African CFA francs. As this subsidy program was fully integrated into the health care system, HELP acted as a third party payer. To ensure the successful implementation of this intervention, other accompanying measures were taken: awareness campaigns for public and community leaders, training and medical supervision of health workers, technical and material support to CSPS, training and support of community health management committees (COGES).

### Sampling process

#### Study sites

Our design follows a matched cohort design for pre-existing community intervention. Indeed, the NGO-based intervention was planned and implemented before the scientific process launched. As the intervention was unfortunately unable to get across all health districts in the Sahel region, the poorest health districts were thus prioritized and prevented us to have a randomized sampling. As a consequence, the present study used a quasi-experimental design that matched intervention health districts to control health districts. The health districts of Sebba and Dori (intervention group with total fee exemption) were matched to the health district of Djibo (control group with 20% remaining fees) based on geo-demographic, socio-cultural characteristics, and public health system levels. For each intervention and control health districts, a total of six CSPS, that best characterized the diversity of the local context, were selected.

#### Sampling population

The study population was defined as women living in one selected intervention or control group and who had delivered in one of the selected CSPS in the 60 days before the date of the interview (March 16 to May 16, 2010). A total of 50 women were randomly selected from the general list provided by each CSPS. Of the 687 and 549 women listed in Sebba and Dori respectively, a total of 299 and 270 were randomly sampled, respectively. In Djibo, a total of 301 women were sampled. Due to limited monetary resources and the lower utilization rate in a user fee context, interviewers were unable to complete the quota of 50 women in each CSPS of Djibo in two months. As a consequence, they were given a second option by extending the 60 days from the review date, remaining within a proper time frame to avoid recall bias. Interviews took place at home.

#### Ethics statement

The Ministry of Health of Burkina Faso examined and approved the ethics component of this research project and authorized the study. Ethical approval was given prior to data collection. A written informed consent for participation in the study was obtained from participants, who were all adults.

### Socio-demographic characteristics

These include characteristics of the mother and her family, such as the woman’s age, parity, ethnic group, matrimonial status, education, occupation, household economic status, and distance from the residence to the nearest health facility. Household economic status was estimated on the value of the selected household’s asset ownership, such as commodities purchased in markets (household assets, e.g. means of transportation, possession of a television, phone), livestock and farmland ownership, household size and housing characteristics (for more details, see a similar study [24]). Household’s economic status was divided into quintiles for better discrimination (from the lowest Q1 to the highest quintile Q5).

### Study instrument

#### Questionnaire

To estimate women’s perception of quality of care, we used a structured questionnaire on post-partum views and opinions, which was adapted from a study done in Senegal [25]. It consisted of 34 questions or items grouped into four topics. Each topic in turn looked at the care provider-patient interaction (traits having to do with explanatory attitude, availability, confidence, carefulness, concern, comfort, and delicacy), conditions of the delivery environment (well-being, comfort, hygiene, and equipment), general comments on nursing care services (human qualities, competence, efficiency, information received, understanding, quality and coordination of care) and barrier factors (delivery care fee, distance from home to CSPS, and honesty of care providers). For each item, the highest satisfaction was given a score of 5 points, the lowest 1 point. The questionnaire was developed in French, translated into local languages and administered by six trained interviewers. A three-day pre-test was carried out. A total of 870 questionnaires were ultimately completed. Complete data were available for 97% of questions. For cases that were missing data for an item, the average for all respondents on the same item in the subscale was used as the response to the missing item.

### Psychometric analyses

To measure women’s perceptions, we used a principal component analysis (PCA) that allows all items to be considered simultaneously and then explored the structure of the quality of care responses across all control-intervention groups. Since items from the same category of delivery care responses are likely to be correlated, the question then arises whether these items could somehow be reduced to fewer items that capture as much as possible of the variation in the original data. The meaning of each dimension is verified on the basis that items selected on the same dimension belong *a priori* to the same category of delivery care responses. For each category, one dimension (component) is created from the weighted linear combination of the retained items and thus provides a composite measure (index) of women’s perceptions.

The Kaiser-Meyer-Olkin measure of sampling adequacy and a significant Bartlett’s test of sphericity confirmed that the data set was appropriate for PCA. Dimensionality was assessed using truncated PCA with varimax rotation. The reliability of each of the selected items on each dimension was evaluated by Cronbach’s scores. Items were retained if they met a criterion of 0.40 for factor loading. We examined the internal consistency reliability of each dimension via Cronbach’s alpha procedures, evaluating the inter-item correlations (0.30-0.70), corrected item-to-total-score correlation (≥ 0.30), and alpha-if-item-deleted (increase of 0.01). A Cronbach’s alpha coefficient > 0.80 was preferentially anticipated for each subscale. The discriminating validity between two indices was demonstrated if the correlation coefficient was less than one minus two times the standard error of the correlation coefficient. For each dimension, responses to the retained items were weighted and averaged to create an overall score for patient care quality and thus a satisfaction index for each group of delivery care responses. For each satisfaction index, women were classified into quintiles (from the lowest, Q1, to the highest quintile, Q5) independently of which fee exemption group they belonged to. The satisfaction index was used as a continuous and categorical variable (quintiles).

To verify that the new PCA-based indices were not associated with the intervention or control group, some multivariate regression analyses were carried out after controlling for all the socio-demographic variables.

Data were entered and analyzed using SPSS statistical software (version 19.0; SPSS, Inc, Chicago, IL). Differences between frequencies of the control and intervention groups (Chi-square tests) were considered statistically significant when p ≤ 0.05.

## Results

### Descriptive results

Tables 1 and 2 show respectively the regional characteristics and socio-demographic characteristics of women in the control and intervention groups.

**Table 1:** Distribution of demographic and public health data characteristics between control and intervention health districts

	**Control**	**Intervention**
	**(Djibo)**	**(Sebba/Dori)**
**Geo-demographic data**		
Surface area (km2)	12 273	13871
Total population in 2010	389 839	474846
% of population below the poverty line in 2003	37.2	52.0
**Socio-cultural characteristics**		
Most practiced religion	Islam	Islam
Most represented ethnic group	Peulh	Peulh
**Public health data in 2010**		
Distribution of health system levels	1 DH, 30 CSPS, 1 MC, 3 PHC	1 DH, 1 RH, 29 CSPS, 1 MC
% of institutional deliveries	59.9%	71.2%

Abbreviations: RH, Regional Hospital; DH, District Hospital; CSPS, Health and Social Promotion Centres; MC, military clinic; PC, private health centre.

*Statistical directory 2010 Ministry of Health - General Secretariat, General Direction for Information and Health Statistics in Burkina Faso.

**Table 2:** Socio-demographic characteristics of women interviewed in the two study groups

	**Control group**	**Intervention group**
	**(with fees)**	**(without fees)**
	N(%)	N(%)
**Age reproductive group**		
≤ 16 yrs	11 (4.1%)	26 (4.4%)
17-34 yrs	241 (89.6%)	514 (86.1%)
≥ 35 yrs	17 (6.3%)	57 (9.5%)
**Number of births**		
Primiparous	68 (25.58%)	161 (26.88%)
2-4	144 (53.53%)	345 (57.60%)
≥ 5	57 (21.19%)	93 (15.53%)
**Ethnic group**		
Peul	75 (27.8%)	480 (80%)
Gurma	1 (0.4%)	70 (11.7%)
Mossi	81 (30%)	22 (3.7%)
Fulsé	66 (24.4%)	0 (0%)
Bella	5 (1.9%)	21 (3.5%)
Other	42 (15.6%)	7 (1.2%)
**Matrimonial status**		
Monogamous	208 (77%)	490 (81.8%)
Polygamous	62 (23%)	101 (16.9%)
Other	0 (0%)	8 (1.4%)
**Educational level completed**		
None	245 (91.1%)	494 (82.6%)
Literate	14 (5.2%)	61 (10.2%)
Primary	7 (2.6%)	31 (5.2%)
Secondary (and +)	3 (1.1%)	12 (2.0%)
**Occupation**		
Farming	253 (94.1%)	493 (82.7%)
Housewife	11 (4.1%)	75 (12.6%)
Other	5 (1.8%)	28 (4.7%)
**Wealth quintiles**		
(from lowest to highest)		
Q1	44 (16.30%)	130 (21.67%)
Q2	33 (12.22%)	141 (23.50%)
Q3	57 (21.11%)	117 (19.50%)
Q4	55 (20.37%)	119 (19.83%)
Q5	81 (30%)	93 (15.50%)
**Distance to nearest health facility**		
0-5 km	185 (68.5%)	353 (59.0%)
6-10 km	60 (22.2%)	152 (25.4%)
11-15 km	14 (5.2%)	53 (8.9%)
>16 km	11 (4.1%)	40 (6.7%)

At each item level, women generally expressed a high level of satisfaction with delivery care in both the intervention and control groups. A total of 90% were satisfied (median of 4) or very satisfied (median of 5). The three items women were less pleased with were: (i) health workers did not sufficiently explain the birth process (median = 2); (ii) a lack of attention at the time of breastfeeding (median = 2); and (iii) the presence of a family member during childbirth was not tolerated (median = 2). Although women in the intervention group were more satisfied with 16 items than women in the control group (non-parametric median test p ≤ 0.05), the latter were more satisfied with the time devoted to them during delivery.

### Satisfaction indices

Among the 34 items, a varimax rotation revealed that a total of 16 items of women’s perception of quality of delivery care loaded satisfactorily on a three-dimension PCA (Table 3). The first dimension of the PCA was as an indicator dealing with quality in care provider-patient interaction screening, particularly staff attitudes and behaviours, the second dimension related to the quality of nursing care services, and the third dimension was an indicator of birth delivery environment. An index was calculated from each of the three dimensions presented in Table 3.

**Table 3:** Characteristics of the three-dimension principal component analysis (factorial loading of selected items and Cronbach’s alpha values on each dimension)

		**Factorial loading of items selected on each dimension**		
	**List of significant items**	**Dimension 1**	**Dimension 2**	**Dimension 3**
		**(21.03%)**	**(19.28%)**	**(12.49%)**
		**Cronbach’s α = 0.81**	**Cronbach’s α = 0.78**	**Cronbach’s α = 0.66**
Care provider-patient interaction	The midwife or nurse showed up nicely for me.	0.701		
	She/he was available to me.	0.683		
	She/he answered all my questions.	0.684		
	She/he explained the labour process and/or childbirth.	0.699		
	She/he was attentive to me and my requests.	0.611		
	She/he stayed with me during labour and/or childbirth.	0.586		
	She/he showed significant human qualities.	0.658		
Quality of nursing care services	I felt confident and safe with her or him.		0.630	
	The midwife or nurse was attentive to my baby.		0.653	
	She/he reassured me about my concerns.		0.661	
	She/he was concerned about my pain.		0.641	
	The midwife or nurse made sure that my baby and I were doing well.		0.772	
	Quality of care was better than anticipated.		0.589	
Environment to quality of birth to delivery environment	I was comfortably installed.			0.682
	The delivery room was clean and hygiene was satisfactory.			0.770
	The temperature in the delivery room was satisfactory.			0.748

From a multivariate linear regression with each satisfaction index as a separate outcome, Table 4 shows that none of the three satisfaction indices was significantly associated with the intervention or control group, even after controlling for all the socio-demographic variables presented in Table 2. The distribution of ratings for the care provider-patient interaction satisfaction index was similar between the intervention and control groups (not shown here). Although not significant, ratings for satisfaction with nursing care services tended to be higher in the intervention group, and lower for the birth delivery environment in the control group.

**Table 4:** Estimates and significance of covariates in the multivariate linear regressions with each separate satisfaction index as outcome

**Variables**	**Index 1**		**Index 2**		**Index 3**	
	**B**	**P value**	**B**	**P value**	**B**	**P value**
Intervention/control	0.232	0.436	0.138	0.506	0.185	0.131
Age reproductive group	0.264	0.428	-0.176	0.449	0.173	0.205
Number of children	-0.086	0.153	0.082	0.051	0.052	0.038*
Ethnic group	0.008	0.946	0.066	0.416	-0.009	0.844
Matrimonial status	0.142	0.603	0.044	0.819	0.062	0.582
Educational level completed	0.204	0.274	0.455	0.000**	0.288	0.000**
Occupation	0.152	0.314	-0.075	0.479	-0.046	0.461
Wealth quintiles	-0.343	0.007**	0.275	0.002**	-0.072	0.165
Distance to nearest health facility	0.077	0.333	-0.151	0.006**	-0.051	0.120

Abbreviations: Index 1, care provider-patient interaction; Index 2, quality of nursing care services; Index 3, environment to quality of birth to delivery.

Significance level *p < 0.05, **p < 0.01.

Table 5 shows that satisfaction with quality of delivery care was similar between the intervention and control groups on all three dimensions.

**Table 5:** Characteristics of average satisfaction and comparison tests between intervention and control groups.

	**Satisfaction index (average)**					
	**Control with fees**			**Intervention without fees**		
Delivery care fee	Very poor	Wealthy	Comparison test (T test)	Very poor	Wealthy	Comparison test (T test)
Nurses with caring attitudes and behaviours	18.48	17.45	0.842	17.23	17.87	0.108
Nursing care services	17.93	16.85	0.451	17.29	16.68	0.929
Delivery environment	9.64	8.88	0.722	9.26	9.21	0.617

### Quintiles of satisfaction

**Figure 1:**
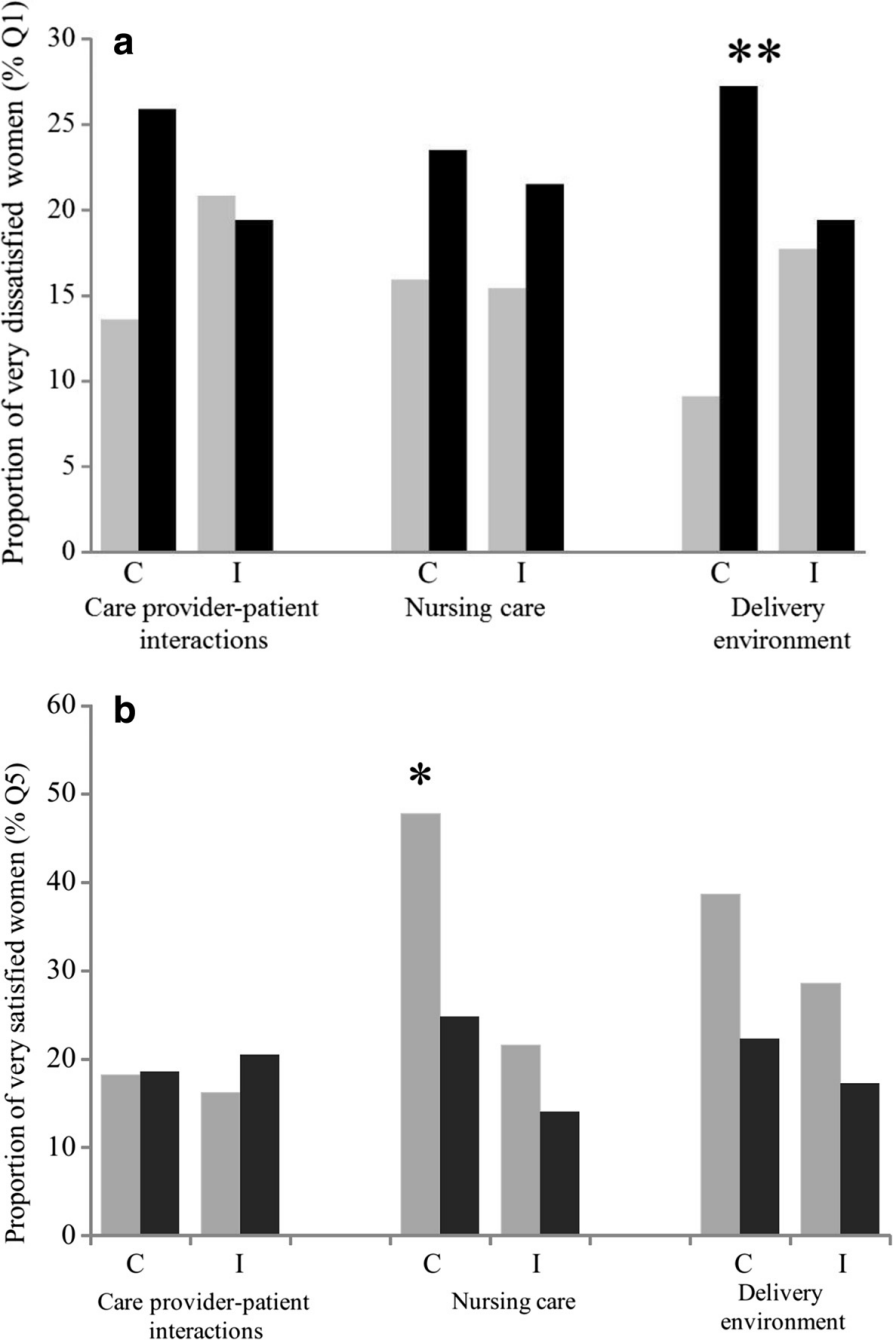
**Frequency of very dissatisfied and satisfied women among the poorest and the wealthiest ones, for each group (control/intervention) and each dimension. a**. Most dissatisfied women and wealth. Frequency of very dissatisfied women (lowest quintile of satisfaction Q1) in the poorest and wealthiest wealth quintile for each group (control/intervention) and each dimension. **b**. Most satisfied women and wealth. Frequency of very satisfied women (highest quintile of satisfaction Q5) in the poorest and wealthiest wealth quintile for each group (control/intervention) and each dimension. **Footnote**. Histograms in grey and black corresponded to the poorest wealth quintile and wealthiest wealth quintile, respectively. Abbreviations: C, control health district of Djibo (with fees); I, Intervention health district of Sebba and Dori (without fees). Significance level on comparison of *p < 0.05, **p < 0.01.

Figure 1a shows the distribution of very dissatisfied women (% lowest quintile of satisfaction, Q1) in the lowest and the highest wealth quintile in each control and intervention group. The sole significant difference we observed was in the control group, where the wealthiest women were more dissatisfied with satisfaction index for delivery environment than the poorest ones (likelihood ratio test, p = 0.017). The room was generally reported as not enough clean and the temperature was not unsatisfactory.

Figure 1b shows the distribution of very satisfied women (% highest quintile of satisfaction, Q5) in the poorest quintile and the wealthiest quintile in each group (control/intervention). Our results showed that the poorest women were more highly satisfied with delivery environment than the wealthiest ones (likelihood ratio test, p = 0.049 and 0.05 in intervention and groups respectively), especially concerning hygiene and comfort.

## Discussion

The three dimensions that were captured from a PCA analysis corresponded to those of some other studies [26,27] and supported our findings. Patients are often inclined by courtesy to respond positively to questions on satisfaction with the quality of care received [28]. This level of courtesy is even higher for satisfaction on interpersonal relationships between care providers and patients. This aspect is the most influential aspect of satisfaction in our indices, as seen in the greatest variance of responses. In a complex technical sector like health care, patients are capable of evaluating neither the level of expertise and basic technical competences of neither their care providers nor the structural quality of care. Thus, it is normal that technical or functional quality ratings in patients were generally high and failed to discordance (quality of nursing care). However, the wealthiest women were more likely to be dissatisfied with the physical conditions of the delivery environment, which generally relates to the lack of financial support of the government state rather than the technical capabilities of care providers. The appearance of the health care centre is important, as patients expect a sense of order and discipline in the environment [29].

All the women who were interviewed demonstrated high levels of satisfaction in both the intervention and control groups, with the exception of a few items (3 out of 34). On half of the items, satisfaction was even greater for the women in the intervention group. Another study [30] showed that despite the increased use of birth delivery services in Dori (intervention group) caused by the abolition of fees, the number of health workers exceeded the number required to conduct delivery activities. Moreover, the fact that the overall satisfaction of delivery care was evaluated three years after the introduction of total fee exemption points to the sustainable effect of that intervention. It would be too ambitious to say that total fee exemption is the only factor responsible for a greater (although not significant) satisfaction. These results suggest that insofar as a substantial financial burden is relieved with total fee exemption at the point of use, while being integrated into a functioning health care system, including community awareness, quality of health workers and infrastructure, quality of care should not be affected, patients’ needs are met, and thus satisfaction is guaranteed. These findings are consistent with other studies showing that greater access to health care is generally associated with increased service utilization and reduced costs for households and more positive impressions [31]. However, this certainly cannot be generalized to all countries in West Africa, as the number of care providers is generally higher in Burkina Faso than observed in neighbouring countries such as Niger and Mali, which respectively had two and five times fewer nurses and midwives than Burkina Faso (mean 7.3 per 10,000 inhabitants) [32].

Total delivery fee exemption seems to benefit all categories of wealth without reinforcing inequalities between very poor and wealthy women in relation to access to delivery care quality, as found in another study in Burkina Faso [31]. Interestingly, the wealthiest women were even more dissatisfied with the delivery environment than the poorest ones, whereas the poorest women demonstrated higher satisfaction with nursing care. The fact that very few, if any, private health centres exist in the study region may partly explain the tendency for the wealthiest women to be less pleased than the poorest ones, as their needs or expectations cannot be met, especially when they contribute to the fees. However, it is interesting to note that in general, the wealthiest women were more likely to be very dissatisfied than the poorest ones, and on the contrary, the poorest women demonstrated higher satisfaction than the wealthiest women.

The quasi-experimental design approach of intervention versus control health districts adopted in the present study may suffer from a number of limitations, and therefore our results must be interpreted cautiously. This survey has introduced a potential for bias from the realities of sampling. Several households were not sampled due to limited access caused by flooding and/or the remoteness of some hamlets. Some bias may also have originated from hypothetical intimidation by the male interviewer of some women. It also remains possible that the interpretation of questionnaires was different between patients, which may affect the generalizability of our findings. Finally, the errors or omissions in the lists taken from the records of the primary health care facilities were beyond our control.

## Conclusion

Despite the recommendations of the African Union [1], abolition of delivery fees is not unanimous, especially among the policy makers in West Africa. Beyond the issues of funding such a policy, it is often the same principle that is denounced, i.e. some going so far as to say that abolition of delivery fees would encourage births in Burkina Faso. While several studies in Burkina Faso [10,31] have shown that total fee exemption increased skilled deliveries at health facilities, which is an essential element of the fight against maternal mortality, the present study demonstrated that there was no difference in perceived quality of care. The effectiveness of that principle having been proved, it would certainly help to make it nationwide and fulfill the commitments made by the head of state of Burkina Faso, who announced in 2010 that financial barriers to access to maternal health care would be removed.

## Competing interest

There is none competing interest between co-authors.

V.R. conducts consultations for NGOs that implement programs for user fee exemption of care.

## Authors’ contributions

AP, the first author, performed all statistical analyses, supported by VR and PF She wrote the first draft and all the authors read, improved, and approved the final manuscript. VR, the principal investigator, led the overall study. AB coordinated and participated in the collection of data in the field. He set up the wealth quintiles of the study population. PF. participated in the writing of the article, particularly the study design, results and discussion sections. All authors read and approved the final manuscript.

## Pre-publication history

The pre-publication history for this paper can be accessed here:

http://www.biomedcentral.com/1472-6963/14/120/prepub
